# mTOR inhibitor improves testosterone-induced myocardial hypertrophy in hypertensive rats

**DOI:** 10.1530/JOE-21-0284

**Published:** 2021-12-06

**Authors:** Jianshu Chen, Jing Yu, Ruowen Yuan, Ningyin Li, Caie Li, Xiaofang Zhang

**Affiliations:** 1 Lanzhou University Second College of Clinical Medicine, Lanzhou, China; 2 Department of Cardiology, Lanzhou University Second Hospital, Lanzhou, China

**Keywords:** postmenopausal, blood pressure, myocardial hypertrophy, androgen, mTOR inhibitor

## Abstract

Compelling evidence has described that the incidence of hypertension and left ventricular hypertrophy (LVH) in postmenopausal women is significantly increased worldwide. Our team’s previous research identified that androgen was an underlying factor contributing to increased blood pressure and LVH in postmenopausal women. However, little is known about how androgens affect LVH in postmenopausal hypertensive women. The purpose of this study was to evaluate the role of mammalian rapamycin receptor (mTOR) signaling pathway in myocardial hypertrophy in androgen-induced postmenopausal hypertension and whether mTOR inhibitors can protect the myocardium from androgen-induced interference to prevent and treat cardiac hypertrophy. For that, ovariectomized (OVX) spontaneously hypertensive rats (SHR) aged 12 weeks were used to study the effects of testosterone (T 2.85 mg/kg/weekly i.m.) on blood pressure and myocardial tissue. On the basis of antihypertensive therapy (chlorthalidone 8 mg/kg/day ig), the improvement of blood pressure and myocardial hypertrophy in rats treated with different dose gradients of rapamycin (0.8 mg/kg/day vs 1.5 mg/kg/day vs 2 mg/kg/day i.p.) in OVX + estrogen (E 9.6 mg/kg/day, ig) + testosterone group was further evaluated. After testosterone intervention, the OVX female rats exhibited significant increments in the heart weight/tibial length (TL), area of cardiomyocytes and the mRNA expressions of ANP, β-myosin heavy chain and matrix metalloproteinase 9 accompanied by a significant reduction in the uterine weight/TL and tissue inhibitor of metalloproteinase 1. mTOR, ribosomal protein S6 kinase (S6K1), 4E-binding protein 1 (4EBP1) and eukaryotic translation initiation factor 4E in myocardial tissue of OVX + estrogen + testosterone group were expressed at higher levels than those of the other four groups. On the other hand, rapamycin abolished the effects of testosterone-induced cardiac hypertrophy, decreased the systolic and diastolic blood pressure of SHR, and inhibited the activation of mTOR/S6K1/4EBP1 signaling pathway in a concentration-dependent manner. Collectively, these data suggest that the mTOR/S6K1/4EBP1 pathway is an important therapeutic target for the prevention of LVH in postmenopausal hypertensive female rats with high testosterone levels. Our findings also support the standpoint that the mTOR inhibitor, rapamycin, can eliminate testosterone-induced cardiomyocyte hypertrophy.

## Introduction

Hypertension has been recognized as the most blatantly visible risk factor for cardiovascular and cerebrovascular diseases (Wang *et al.* 2020, Zhang *et al.* 2020). It is known that left ventricular hypertrophy (LVH) is one of the most important manifestations of hypertension-mediated organ damage (HMOD) (Cao *et al.* 2019, Yildiz *et al.* 2020). In recent years, compelling evidence has demonstrated that the prevalence of hypertension and LVH in postmenopausal women have increased significantly worldwide (Luczak & Leinwand 2009, Brahmbhatt *et al.* 2019).

There is no doubt that antihypertensive treatment brings benefits to patients with hypertension and LVH (Dahlöf *et al.* 2002). Although the current treatment of hypertension and HMOD has achieved certain results, LVH cannot be reversed to a greater extent even under the premise of reaching the standard for lowering blood pressure. What is more noteworthy is that the occurrence and treatment of LVH in women after menopause are more complicated than those in men (Li *et al.* 2019). Thus, new scientific understanding of the pathogenesis and treatment of postmenopausal hypertensive women and LVH needs to be investigated.

The occurrence of LVH in postmenopausal hypertension patients is closely associated with the changes of sex hormone axis in postmenopausal women (Gohar *et al.* 2020, Shawky *et al.* 2020). Experimental studies have confirmed that dynamic changes in estrogen levels are significantly correlated with blood pressure levels in rats (Guivarc’h *et al.* 2020). However, a number of clinical studies have confirmed that it is not enough to explain the occurrence of postmenopausal hypertension and heart damage from a single estrogen reduction (Grady *et al.* 2002, Grodstein *et al.* 2006). Except for estrogen, changes in androgen levels may contribute to the occurrence of postmenopausal cardiovascular disease. Jiroutek *et al.* reported an increase in serum total testosterone levels in postmenopausal women after 10 years of follow-up (Jiroutek *et al.* 1998). Animal studies have shown that the serum testosterone of elderly female spontaneously hypertensive rats (SHR) is increased by four times compared with younger rats (Fortepiani *et al.* 2003). Our team’s pre-clinical studies also confirmed that testosterone levels in postmenopausal hypertensive women are elevated, and testosoterone plays a potential role in postmenopausal hypertension and HMOD (Li *et al.* 2019, Jianshu *et al.* 2021). Although many studies have explored the role of testosterone in regulating vascular tension (Costa *et al.* 2018, Reckelhoff 2019), few studies have investigated the molecular mechanism of LVH in patients with testosterone-induced postmenopausal hypertension.

Mammalian rapamycin receptor (mTOR) is a potentially important regulatory factor in various regulatory pathways that affect cardiac function (Huang *et al.* 2020, Liu & Sabatini 2020). mTOR inappropriate activation can lead to adverse cardiovascular events (Ferrara-Romeo *et al.* 2020). Aortic constriction-induced myocardial hypertrophy is accompanied by an increase in mTOR activity (McMullen *et al.* 2004). Other studies have also suggested that the expression of mTOR protein in myocardial tissue is increased during exercise-mediated physiological hypertrophy (Zhang *et al.* 2010). Therefore, mTOR is a crucial regulator of the maintenance of cardiac function under myocardial compensatory and pressure overload. These studies led us to propose the hypothesis that the mTOR signaling pathway is involved in testosterone-induced elevated blood pressure and cardiac hypertrophy in postmenopausal women. Thus, in this study, we focused on ovariectomized (OVX) SHR to validate that the mTOR/ribosomal protein S6 kinase (S6K1)/4E-binding protein 1 (4EBP1) pathway was involved in the development of testosterone-induced OVXSHR myocardial hypertrophy. We also investigated that mTOR inhibitors can postpone testosterone-induced OVXSHR cardiac hypertrophy. On the basis of antihypertensive therapy, our findings provide new therapeutic ideas for delaying LVH in patients with postmenopausal hypertension.

## Materials and methods

### Experimental animals

Twelve-week-old female SHR and age-matched Wistar Kyoto (WKY) without specific pathogen free were obtained from Laboratory Animal Center, Medical College of Lanzhou University. All animal handling and operations met the requirements of the guidelines for the Care and Use of Laboratory Animals. This study was approved by the Laboratory Animal Welfare Ethics Committee of the Lanzhou University Second Hospital (Grant Number: D2020-03). The rats were maintained under conditions of controlled temperature (22–25°C), humidity (40–60%), 12 h light:12 h darkness cycle and free access to standard laboratory chow and tap water.

### Model establishment and grouping

Rats were OVX using the previously described method by Lee *et al.* (2008). OVX in rats was performed under pentobarbital anesthesia (50 mg/kg, i.p.). The rats in sham-operated group were only dissected to explore bilateral ovaries without other operation. After the operation, the rats were placed separately and returned to the cage for further feeding after the rats recovered from anesthesia. Postoperative incision was observed regularly for bleeding and infection. Vaginal smears were performed on SHR of sham operation group and OVX 1 week after operation. The sign of successful model establishment was that continuous vaginal shedding cell smear examination showed the disappearance of estrous cycle changes in rats and the cell type was estrous interphase I and II.

We designed two-phased experimental studies:

(一) Female WKY were used as control group. Two weeks after OVX, female SHRs were randomly divided into five groups and intervened for 1 month according to previous studies: (1) sham-operated group; (2) OVX group; (3) OVX + estrogens (E) group; (4) OVX + E + T group (E: 9.6 mg/kg/day, ig; T: 2.85 mg/kg/weekly, i.m.). On the one hand, the dose of drug intervention should be ensured as much as possible at physiologically relevant concentrations, and on the other hand, it is based on literature reviews and pre-experimental results (Araujo *et al.* 2017, Costa *et al.* 2018).
(二) According to the experimental model of group 5 (OVX + E + T) in the first stage experiment, the model was made again and randomly divided into five groups for 3 weeks of intervention according to previous studies and pre-experimental results: (1) low-dose group (chlorthalidone 8 mg/kg/day, ig + rapamycin 0.8 mg/kg/day, i.p.); (2) medium-dose group (chlorthalidone 8 mg/kg/day, ig + rapamycin 1.5 mg/kg/day, i.p.); (3) high-dose group (chlorthalidone 8 mg/kg/day, ig + rapamycin 2 mg/kg/day, i.p.); (4) chlorthalidone group (8 mg/kg/day, ig); (5) vehicle group (2 mg/kg/day, i.p.). The selection of intervention drugs is based on the following: thiazide diuretics is the most classical and earliest antihypertensive drugs, and chlorthalidone is the most commonly used drug as a simple antihypertensive in antihypertensive treatment studies. In addition to the benefits of lowering blood pressure, current studies suggest that the impact on LVH is less than other antihypertensive drugs. The doses of chlorthalidone and rapamycin are based on literature review and pre-experimental results (McMullen *et al.* 2004, Zhou *et al.* 2008).

### Cardiac function and blood pressure analysis

Thoracic echocardiography was performed on rats after being anesthetized with pentobarbital (50 mg/kg i.p.). Two-dimensional images of the long and short axes of the parasternal left ventricle were obtained according to the standard. M-mode echocardiography recordings were obtained for the level of papillary muscle. The main indicators reflecting the heart structure of rats included left atrial (LA) diameter, left ventricular end-diastolic diameter (LVEDD), left ventricular end-systolic diameter, diastolic interventricular septal thickness (IVST), diastolic left ventricular posterior wall thickness (LVPWT) and left ventricular mass (LVM). The main indexes reflecting the cardiac function of rats included left ventricular ejection fraction (LVEF), left ventricular fractional shortening (LVFS), the peak flow velocity of early diastolic of mitral valve (E), the peak flow velocity of late diastolic of mitral valve (A), E/A ratio (E/A), the peak velocity of early diastolic motion of mitral annulus (e’) and E/e’. The awake systolic blood pressure (SBP) and diastolic blood pressure (DBP) were collected at the same time period of week using the BESN-II animal tail artery non-invasive pressure measurement system and BL-420E biological signal acquisition and processing system during drug intervention period. Each group of original data was the means for three consecutive cycles.

### Quantitative real-time PCR (qPCR) analysis

Total RNA was extracted from left ventricular myocardial tissue using Trizol reagent. The mRNA levels of mTOR, 40S ribosomal protein S6K1, eukaryotic initiation factor 4EBP1 in myocardial tissue were quantitatively analyzed by qPCR. GAPDH was used as an internal control. The conditions of qPCR were 95°C for 20 s, 40 cycles at 95°C for 3 s and 60°C for 30 s. All primer sequences were as follows: *Anp* (front TGACAGGATTGGAGCCCAGAG, rear TCGATCGTGATAGATGAAGACAGGA), myosin heavy chain (*Bmhc*) (front CCACAACCAGTA-CAAGTTCG, rear CTGGATGATCAGCAAG-GAGT), *Mtor* (front GCTGATCACAGTAAATGGGCAAGA, rear CCAACAGCTTGCCGTGGATA), *S6k1* (front AAATCCGATCGCCTCGAAGA, rear CACTTGTTTCCATTGGGTATTCCAC), *4Ebp1* (front TCACTAGCCCTACCAGCGATGAG, rear CCAGAAGCATCACTGCGTCCTAT), matrix metalloproteinase 9 (*Mmp9*) (front CCCTGCGTATTTCCATTCATC, rear ACCCCACTTCTTGTCAGCGTC), tissue inhibitor of metalloproteinase 1 (*Timp1*) (front CCCAACCCACCCACAGACAGC, rear AACGGCCCGCGATGAGAAACT) and GAPDH (front TGTGTCCGTCGTGGATCTGA, rear TTGCTGTTGAAGTCGCAGGAG).

### Western blot analysis

Left ventricular tissue was placed in the prepared Radio-Immunoprecipitation Assay (RIPA) lysis buffer with 1 mL. The myocardial tissue was fully grounded to extract the protein. Lowry method was used to determine the total protein content. Proteins were isolated by SDS-PAGE gel and transferred to PVDF membrane. The PVDF membrane was sealed with PBS solution containing 5% skimmed milk powder for 1 h and the excess skimmed milk powder was washed away. After adding the diluted first antibodies of mTOR (1:1000; Cell Signaling Technology, CST2983), S6K1 (1:1000; Cell Signaling Technology, AY4775 CST9202), 4EBP1 (1:1000; Abcam, ab32024), eukaryotic translation initiation factor 4E (eIF4E) (1:800; Thermo Fisher, PA5-86047) and GAPDH (1:7000; Proteintech, Wuhan, China, 60004-1-Ig) to PVDF membrane, the membrane was incubated overnight in a shaking bed at 4°C. After washing the PVDF membrane for three times with TBST, the corresponding diluted secondary antibody was added. After incubation at room temperature for 1 h, the membrane was washed with TBST three times again. ECL chemiluminescence liquid developed luminescence, gel imaging system took pictures, and Image Pro Plus image analysis system analyzed protein bands.

### Histopathological study

Myocardial tissue was fixed in 4% paraformaldehyde solution for more than 24 h. After formaldehyde fixation, paraffin sectioning and deparaffinization, the myocardial tissue was stained with wheat germ agglutinin solution and the nucleus was counterstained with DAPI. After using anti-fluorescence quenching agent to seal the film, the image was collected by fluorescence microscope. The cell nucleus was blue under the excitation of UV and the positive expression was green light. The target area of the slice was selected for 400× imaging. The tissue should be filled with the whole visual field during imaging to ensure the consistent background light of each photograph. The number of cardiomyocytes and visual field area were measured in three visual fields per slice, and the area of individual cardiomyocytes was calculated. Image Pro-Plus 6.0 software was used to analyze the tissue sections.

### Enzyme-linked immunosorbent assay (ELISA) analysis

Abdominal aortic blood was extracted from rats in each experimental group after intervention. After centrifugation at 1000 r.p.m. for 5 min, the plasma was separated and stored in a refrigerator at −80°C. The levels of estradiol and testosterone were determined by ELISA method. At the end of the mTOR inhibitor intervention, urine was collected in the metabolic cage for 24 h. The concentrations of urinary microalbumin were detected by ELISA. All operations were carried out in accordance with the requirements of the kit instructions.

### Statistical analysis

The statistical analysis was performed using SPSS22.0 (SPSS Inc.) statistical software. Shapiro–Wilk’s method tests the normal distribution of data. Continuous variables in accordance with normal distribution were represented by mean ± s.d. Differences between groups were analyzed by one-way ANOVA with Dunnett’s* post hoc* test. Least significant difference test was used for homogeneity of variance, and Tamhan’s test was used for non-homogeneity of variance. Data that did not conform to normal distribution were estimated by Kruskal–-Wallis test and Dunn’s multinomial comparison test. Repeated measures one-way ANOVA was used to compare blood pressure at different time points in the rats. *P* < 0.05 indicated that the difference was statistically significant.

## Results

### Model evaluation and survival status

In fact, a total of 90 SHRs were reared, 10 of which underwent sham operation, and 77 of the other 80 OVX rats survived. Vaginal smear was taken continuously for 5 days starting 1 week after the operation in sham-operated and OVX group. The cell types in the OVX group were estrous interphase I and estrous interphase II, which proved that the establishment of postmenopausal animal model was successful. In contrast, the cell types of WKY and sham-operated group belonged to pre-estrus or estrus (Supplementary Fig. 1, see section on supplementary materials given at the end of this article).

### OVX regulated SHR blood pressure, cardiac structure and related gene expression

**Figure 1:**
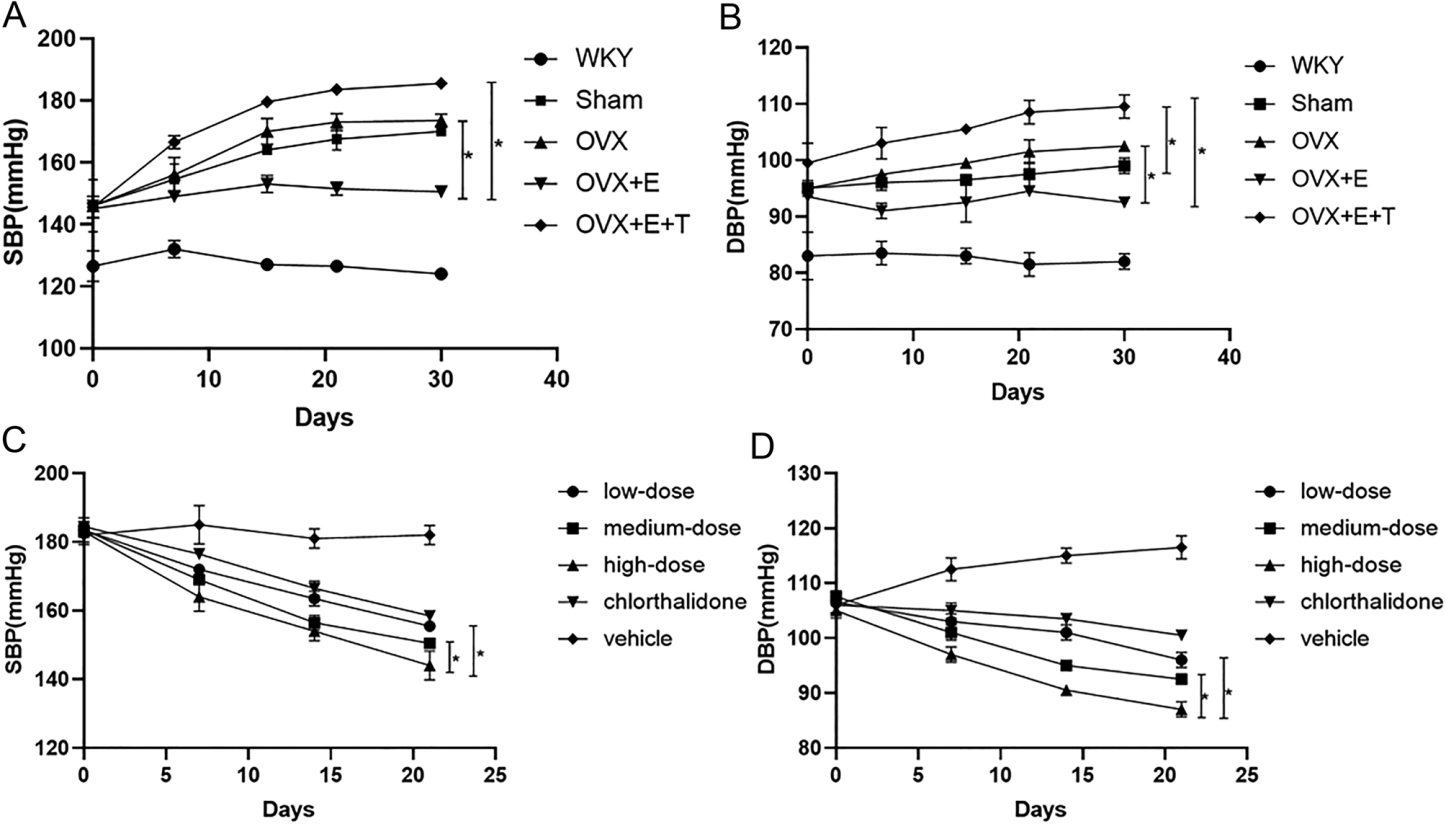
mTOR inhibitor rapamycin abolished the effect of testosterone on OVX SHR blood pressure elevation. (A and B) Dynamic evolution of SBP and DBP level in each group at 30 days. (C and D) Effects of different dose gradients of rapamycin on SBP and DBP in OVX SHR induced by T on the basis of antihypertensive therapy. Data shown are expressed as mean ± S.E.M. The *P* values were determined by one-way ANOVA and repeated measures one-way ANOVA (*n* = 6 independent biological samples). *P < 0.05. DBP, diastolic blood pressure; E, estrogen ; mTOR, mammalian rapamycin receptor; OVX, ovariectomized; SBP, systolic blood pressure; SHR, spontaneously hypertensive rats; T, testosterone; WKY, Wistar Kyoto.

**Figure 2:**
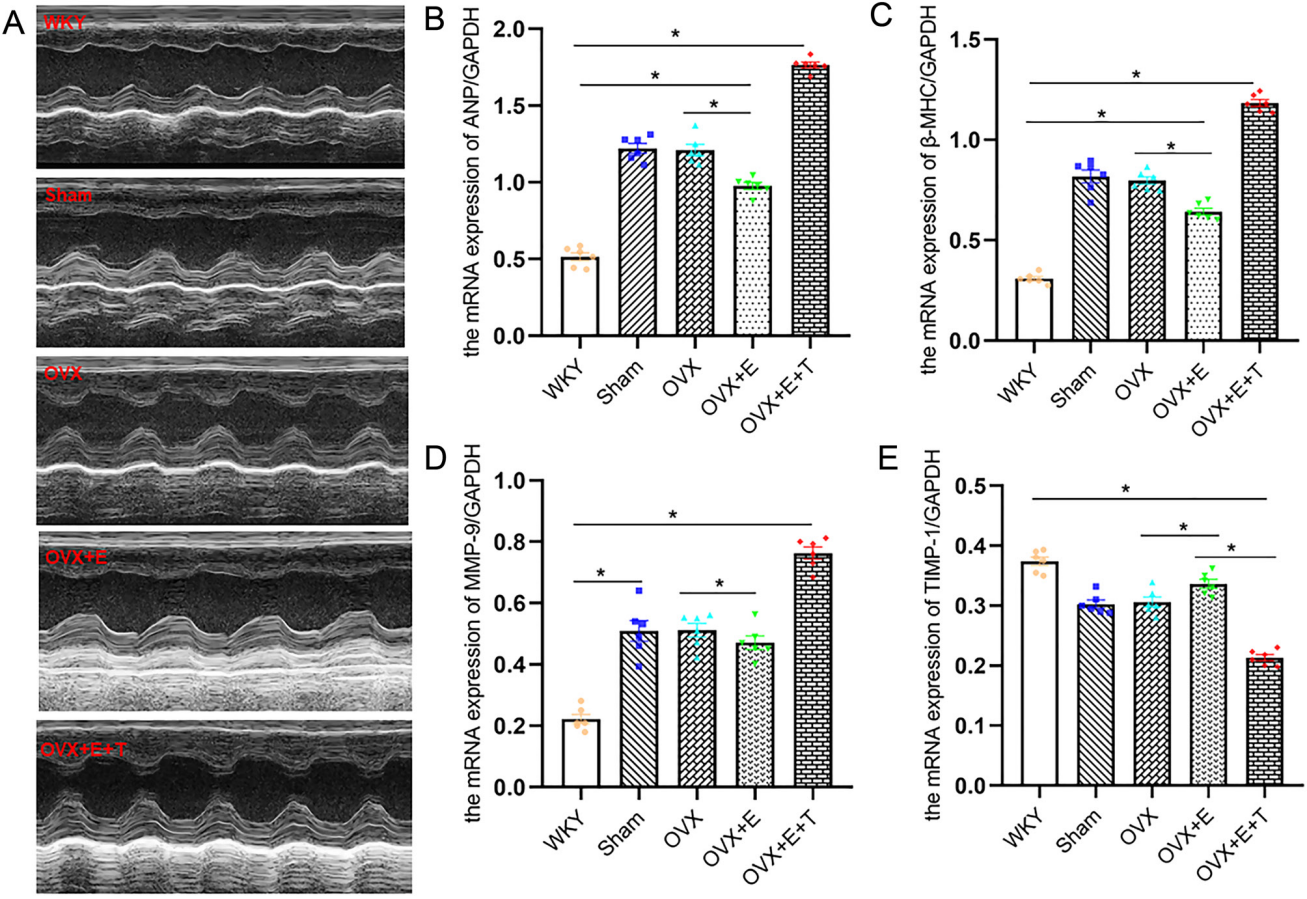
Testosterone induces OVX SHR myocardial hypertrophy and hypertrophy-related gene expression. (A) T augmented the diastolic interventricular septal thickness, diastolic left ventricular posterior wall thickness and left ventricular mass of OVX SHR. (B, C, D, and E) mRNA expression of β-MHC, ANP, MMP-9 and TIMP-1 by real-time RT-PCR. Data shown are expressed as mean ± S.E.M. The *P* values were determined by one-way ANOVA (*n* = 6 independent biological samples). *P < 0.05. E, estrogen; MHC, myosin heavy chain; MMP, matrix metalloproteinase; OVX, ovariectomized; SHR, spontaneously hypertensive rats; T, testosterone; TIMP, the tissue inhibitor of metalloproteinase; WKY, Wistar Kyoto.

**Figure 3:**
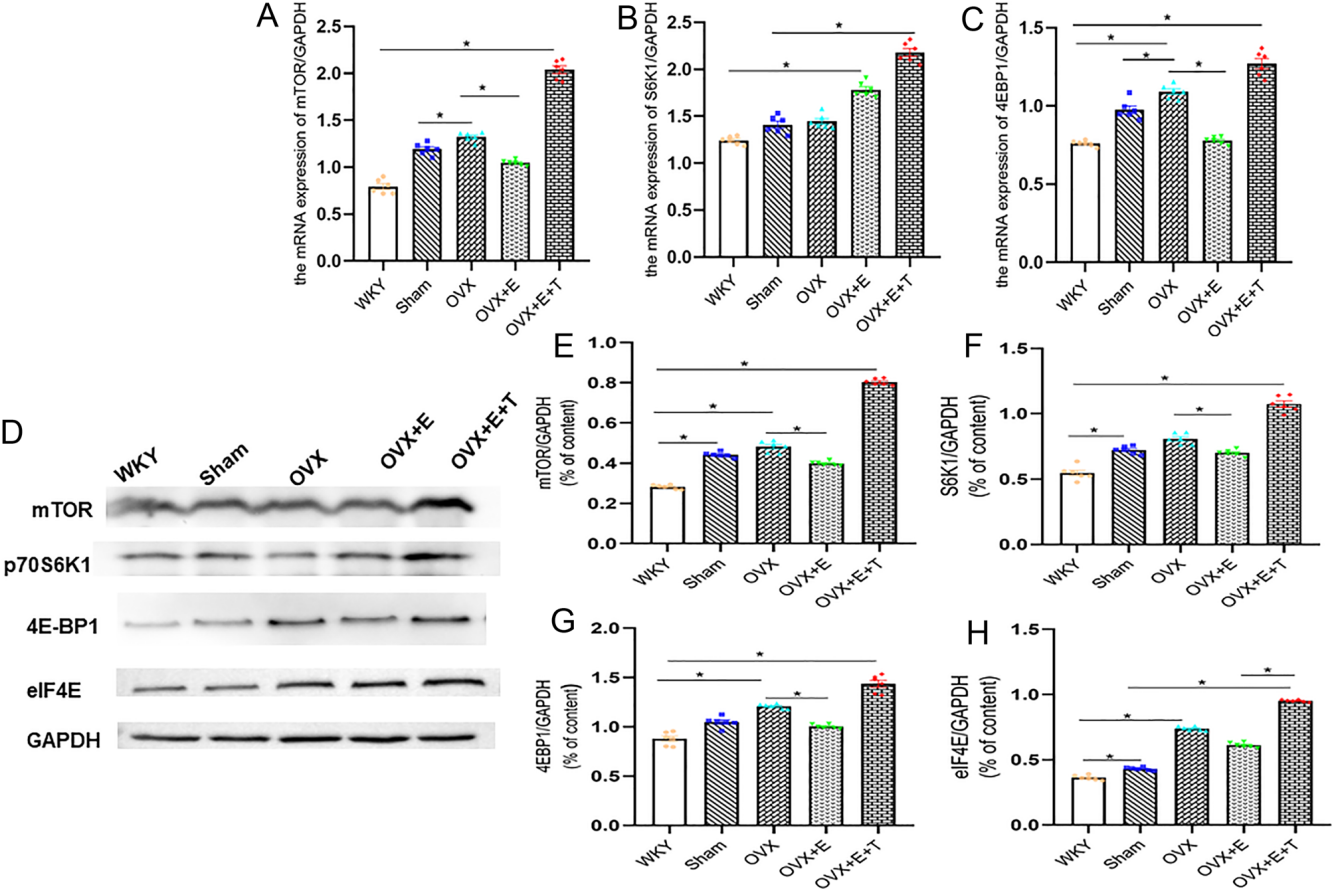
Testosterone aggrandized the expression level of mTOR signaling pathway protein and mRNA in OVX SHR myocardial tissue. (A, B and C) T induced the mRNA expression levels of mTOR, S6K1 and 4EBP1 in the myocardial tissue. (D, E, F, G, and H) T increased the expression level of mTOR/S6K1/4EBP1/eIF4E signaling pathway protein in OVX SHR myocardial tissue. Data shown are expressed as mean ± S.E.M. The *P* values were determined by one-way ANOVA (*n* = 6 independent biological samples). *P < 0.05. E, estrogen; eIF4E, eukaryotic translation initiation factor 4E; mTOR, mammalian rapamycin receptor; OVX, ovariectomized; S6K1, ribosomal protein S6 kinase; T, testosterone; WKY, Wistar Kyoto; 4EBP1, 4E-binding protein 1.

**Figure 4:**
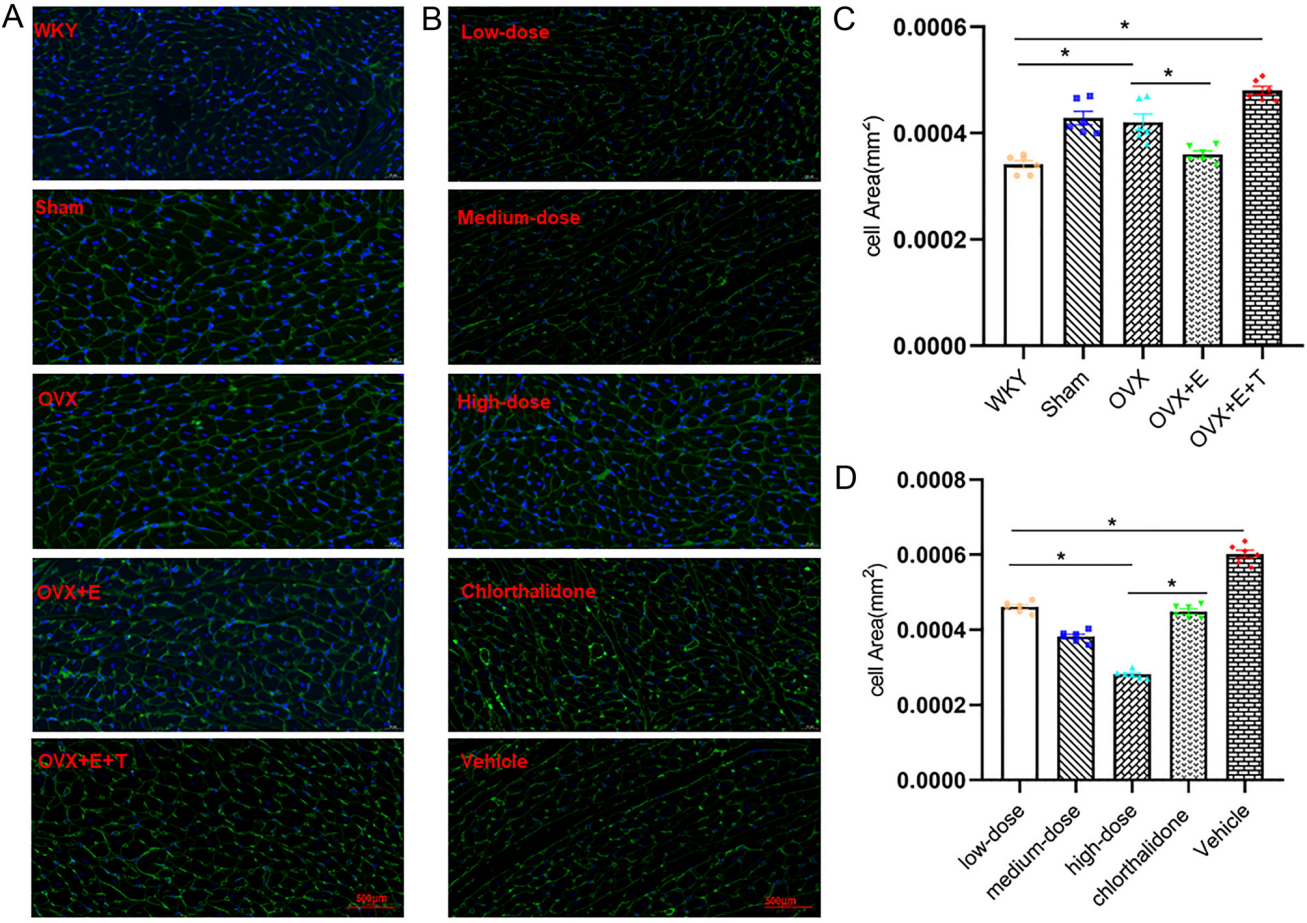
Rapamycin eliminated the effect of T on myocardial area (original magnification ×400). (A and B) WGA staining results in myocardial tissue of each group. (C) T enhanced the myocardial area of OVX SHR. (D) Rapamycin abolished the effect of T on myocardial area. Data shown are expressed as mean ± S.E.M. The *P* values were determined by one-way ANOVA (*n* = 6 independent biological samples). *P < 0.05. E, estrogen; OVX, ovariectomized; T, testosterone; WGA, wheat germ agglutinin; WKY, Wistar Kyoto.

Heart weight (HW) and HW/tibial length (TL) were significantly increased after OVX in SHR. Compared with WKY group, OVX and sham-operated group significantly reduced the ratio of uterine weight (UW) to TL. Plasma E and T levels in OVX group were significantly lower than those in WKY and sham group (Table 1). The SBP and DBP levels did not change significantly in the OVX group (Fig. 1A and B). The LVM level of WKY group (334.67 ± 5.68 g) was lower than that in the other four groups (sham 402.96 ± 6.91 g vs OVX 390.54 ± 9.12 g vs OVX + E 357.69 ± 5.21 g vs OVX + E + T 486.98 ± 2.68 g). There was no significant statistical difference in LVEDD, EF and LVFS among all groups (Fig. 2A and Table 2). The dissolution curves of mRNA showed a single peak curve, which indicated that the PCR product was free from contamination and has good specificity of amplification reaction. The mRNA expression of mTOR and 4EBP1 increased after OVX in SHR, while there was no difference in the expression of S6K1, ANP, β-MHC, MMP-9 and TIMP-1 (Figs 2B, C, D, E and  3A, B, C). mTOR, 4EBP1 and eIF4E expression was augmented by OVX in comparison to sham group (Fig. 3D, E, G and H). However, the protein expression levels of S6K1 in myocardial tissue were different from the same pattern of mTOR protein expression. There was no difference in S6K1 protein expression between the sham-operated and OVX (Fig. 3F). There was also no statistical difference in the area of myocardial cells between the sham-operated group and the OVX group (Fig. 4A and C).

**Table 1:** General features of experimental animals. According to the experimental model of group 5 (OVX + E + T) in the first stage experiment, the model was made again for the second phased experimental. Results are expressed as mean ± s.e.m. of six animals/group. The *P* values were determined by one-way ANOVA.

	BW (g)	HW (mg)	HW/BW (mg/g)	UW (mg)	TL (mm)	HW/TL (mg/mm)	UW/TL (mg/mm)	E (pg/mL)	T (ng/mL)
Experiment 1									
WKY	204.25 ± 10.90	564.3 ± 11.78	2.77 ± 0.11	471.40 ± 15.99	37.75 ± 2.06	14.97 ± 0.61	12.51 ± 0.54	18.28 ± 0.49	15.07 ± 0.42
Sham	202.50 ± 0.71	680.45 ± 22.83^a^	3.36 ± 0.13^a^	382.95 ± 12.37^a^	38.00 ± 2.83	17.93 ± 0.72^a^	10.09 ± 0.42^a^	18.80 ± 1.41	14.96 ± 0.62
OVX	215.50 ± 3.54	728.75 ± 34.86^a,^ ^b^	3.24 ± 0.12^a^	259.80 ± 19.94^a^ ^,b^	35.85 ± 0.21	20.32 ± 0.86^a,b^	7.25 ± 0.59^a,b^	11.75 ± 0.93^a,b^	13.14 ± 0.12^a,b^
OVX + E	212.00 ± 11.63	651.37 ± 12.84^a^ ^,c^	3.07 ± 0.11^a,b^	246.22 ± 18.39^a,b^	37.75 ± 1.71	17.27 ± 0.69^a,c^	6.52 ± 0.48^a,b^	18.59 ± 0.79^c^	13.68 ± 0.45^a,b^
OVX + E + T	207.25 ± 10.24	779.05 ± 18.46^a,b,c,d^	3.76 ± 0.15^a,b^ ^,c,d^	262.45 ± 30.95^a,b^	35.00 ± 1.63	22.28 ± 1.54^a,b,c^ ^,d^	7.51 ± 0.97^a,b^	17.85 ± 0.55^c^	37.18 ± 0.77^a,b,c,d^
Experiment 2									
Low-dose	229.00 ± 4.42	730.50 ± 1.27	3.19 ± 0.06	269.90 ± 6.64	39.50 ± 0.71	18.37 ± 0.53	6.83 ± 0.04	17.60 ± 0.11	37.76 ± 0.63
Medium-dose	234.50 ± 5.71	714.70 ± 5.51^e^	3.05 ± 0.01^e^	261.05 ± 4.87	38.00 ± 1.41	18.56 ± 0.54	6.87 ± 0.12	18.11 ± 0.79	36.61 ± 0.21
High-dose	239.50 ± 5.19	664.10 ± 2.40^e,f^	2.27 ± 0.09^e,f^	260.75 ± 5.16	41.00 ± 1.25	16.21 ± 0.11^e,f^	6.36 ± 0.09	18.30 ± 0.71	36.46 ± 1.49
Chlorthalidone	228.50 ± 4.95	725.20 ± 2.26^g^	3.29 ± 0.04^f,g^	279.55 ± 4.03	40.00 ± 0.15	18.13 ± 0.56^g^	6.90 ± 0.02	18.25 ± 1.18	37.51 ± 0.72
Vehicle	220.00 ± 4.41	813.50 ± 1.73^e^ ^,f,g,h^	3.78 ± 0.12^e,f,g,h^	263.55 ± 7.74	40.50 ± 0.71	20.09 ± 0.78^e,f,g,h^	6.59 ± 0.43	18.59 ± 1.36	36.14 ± 1.53

^a^*P* < 0.05 vs WKY; ^b^*P* < 0.05 vs sham; ^c^*P* < 0.05 vs OVX; ^d^*P* < 0.05 vs OVX + E; ^e^*P* < 0.05 vs low dose; ^f^*P* < 0.05 vs medium dose; ^g^P < 0.05 vs high dose; ^h^P < 0.05 vs chlorthalidone.

BW, body weight; E, estrogen; HW, heart weight; OVX, ovariectomized; TL, tibial length; T, testosterone; UW, uterine weight; WKY, Wistar Kyoto.

**Table 2:** Echocardiographic parameters of each group were compared to evaluate the cardiac structure and function. Results are expressed as mean ± s.e.m. of six animals/group. The *P* values were determined by one-way ANOVA.

	LVEDD (mm)	LVESD (mm)	IVSD (mm)	LVPWTD (mm)	LVM (g)	LA (mm)	EF (%)	FS (%)	E/A	E/e’
Experiment 1										
WKY	4.62 ± 0.06	2.71 ± 0.34	1.42 ± 0.01	1.49 ± 0.04	334.67 ± 5.68	2.84 ± 0.17	78.00 ± 7.07	41.00 ± 7.07	0.56 ± 0.02	0.37 ± 0.01
Sham	4.80 ± 0.03	3.72 ± 0.58	2.55 ± 0.01^a^	2.54 ± 0.04^a^	402.96 ± 6.91^a^	3.14 ± 0.09^a^	76.00 ± 1.41	37.00 ± 1.14	0.59 ± 0.01	0.32 ± 0.02
OVX	4.63 ± 0.08	2.58 ± 0.81	2.55 ± 0.04^a^	2.58 ± 0.06^a^	390.54 ± 9.12^a^	3.09 ± 0.07^a^	80.00 ± 1.42	40.00 ± 2.83	0.56 ± 0.01	0.33 ± 0.03
OVX + E	4.57 ± 0.06	3.03 ± 0.06	2.38 ± 0.01^a^ ^,b,c^	2.49 ± 0.09^a^	357.69 ± 5.21^b,c^	2.91 ± 0.03	73.00 ± 2.83	39.00 ± 1.41	0.59 ± 0.03	0.24 ± 0.02^a,b,c^
OVX + E + T	4.70 ± 0.19	2.62 ± 0.14	2.65 ± 0.06^a,b,c,d^	2.76 ± 0.04^a,b,c,d^	486.98 ± 2.68^a,b,c,d^	3.38 ± 0.06^a,c,d^	72.00 ± 4.24	40.00 ± 2.83	0.69 ± 0.02^a,b,c,d^	0.44 ± 0.02^a,b,c,d^
Experiment 2										
Low dose	5.26 ± 0.17	3.21 ± 0.12	2.56 ± 0.01	2.48 ± 0.07	440.59 ± 16.83	2.99 ± 0.12	75.5 ± 4.95	47.00 ± 1.21	0.64 ± 0.08	0.45 ± 0.07
Medium dose	5.27 ± 0.73	2.54 ± 1.13	2.43 ± 0.01	2.42 ± 0.09	350.78 ± 17.54^e^	2.95 ± 0.13	72.00 ± 0.02	47.50 ± 2.12	0.66 ± 0.08	0.39 ± 0.03
High dose	5.06 ± 0.26	3.12 ± 0.79	1.13 ± 0.18^e,f^	1.22 ± 0.06^e,f^	263.12 ± 3.20^e,f^	2.94 ± 0.08	76.50 ± 2.12	48.00 ± 1.41	0.47 ± 0.02^e,f^	0.25 ± 0.04^e,f^
Chlorthalidone	5.14 ± 0.28	3.22 ± 0.09	2.55 ± 0.04^g^	2.49 ± 0.09^g^	443.86 ± 20.17^f,g^	3.08 ± 0.10	71.50 ± 1.12	44.50 ± 1.57	0.63 ± 0.03^g^	0.45 ± 0.03^g^
Vehicle	5.53 ± 0.15	3.54 ± 0.28	2.67 ± 0.03^f,g^	2.80 ± 0.03^e,f,g,h^	530.51 ± 10.98^e,f,g,h^	3.38 ± 0.06^e,f,g,h^	74.50 ± 0.71	44.00 ± 1.30	1.34 ± 0.05^e,f,g,h^	0.58 ± 0.02^e,f,g,h^

^a^*P* < 0.05 vs WKY; ^b^*P* < 0.05 vs sham; ^c^*P* < 0.05 vs OVX; ^d^*P* < 0.05 vs OVX + E; ^e^*P* < 0.05 vs low dose; ^f^*P* < 0.05 vs medium dose; ^g^*P* < 0.05 vs high dose; ^h^*P* < 0.05 vs chlorthalidone.

E, estrogen; E/A, peak velocity during early filling (E), late filling from atrial contraction (A); EF, ejection fraction; FS, fractional shortening; IVST, interventricular septal thickness; LA, left atrial; LVEDD, left ventricular end-diastolic diameter; LVESD, left ventricular end-systolic diameter; LVM, left ventricular mass; LVPWT, left ventricular posterior wall thickness; OVX, ovariectomized; WKY, Wistar Kyoto.

### Estrogen was involved in OVX SHR blood pressure regulation, cardiac hypertrophy and related gene expression

HW and HW/TL in OVX + E group were lower than those in OVX group. Plasma estrogen levels were increased after ovariectomy with SHR with E supplementation (Table 1). Before drug intervention, the baseline level of blood pressure levels was same in each group. The SBP level of OVX + E group was lower than that of OVX group at the end of day 30 of intervention (Fig. 1A and B). Diastolic IVST OVX + E group was significantly lessened in comparison to those of sham-operated and OVX group (Fig. 2A and Table 2). Induction of estrogen alone reduced the mRNA expression levels of *Anp*, *Bmhc* and MMP-1 in myocardial tissue isolated from OVX SHR, while *Timp1* was significantly decreased (Fig. 2B, C, D and E). The levels of *Mtor*, *S6k1*, *4Ebp1* and eIF4E were reduced in myocardial tissue of OVX + E group compared to those of OVX group (Fig. 3D, E, F, G and H). Induction of E alone reduced the area of myocardial cells isolated from OVX SHR. However, it was difficult to restore the area of myocardial cells to the level of WKY rats under the protection of estrogen (Fig. 4A and C).

### Testosterone induced OVX SHR blood pressure elevation, cardiac hypertrophy and mTOR related gene expression

The association of testosterone to estrogen abolished the effect of estrogen. After testosterone intervention, the OVX female rats exhibited significant increments in the HW/TL, HW/body weight (BW) and area of cardiomyocytes accompanied by a significant increase in the serum testosterone levels. The testosterone level in OVX + E + T group was higher than that in other groups (Table 1). Compared with OVX + E group, the levels of SBP and DBP in OVX + E + T group increased at the end of the 30th day. There were statistically significant differences in blood pressure at various time points after testosterone intervention, and the effect of testosterone on blood pressure remained stable (Fig. 1A and B). The levels of IVST, LVPWT, LVM, E/A, and E/e’ in the OVX **+** E + T group were greater than those in the OVX + E group (Fig. 2A and Table 2). The qPCR results showed that the mRNA expression levels of ANP, β-MHC and MMP-9 in the myocardial tissue of the OVX + E + T group increased in comparison to the sham-operated, OVX and OVX+E group accompanied by a significant reduction in the TIMP-1 (Fig. 2B, C, D and E). The protein expression levels of mTOR, S6K1, 4EBP1 and eIF4E were augmented in myocardial tissue of OVX + E + T group compared to the other four groups (Fig. 3D, E, F, G and H).

### mTOR inhibitor rapamycin abolished the effect of T on OVX SHR blood pressure elevation

At the end of day 14 and 21, the high-dose rapamycin reduced significantly SBP and DBP levels when compared to the low-dose and medium-dose rapamycin. There was no difference in SBP and DBP levels between the medium-dose group and the low-dose group at the end of day 21 after intervention. After the intervention of different doses of rapamycin, the blood pressure measurement values of rats at various time points had statistical difference, and the effect of rapamycin on blood pressure remained stable (Fig. 1C and D).

### mTOR inhibitor rapamycin alleviated the effect of T on cardiac hypertrophy and related gene expression in OVX SHR

**Figure 5:**
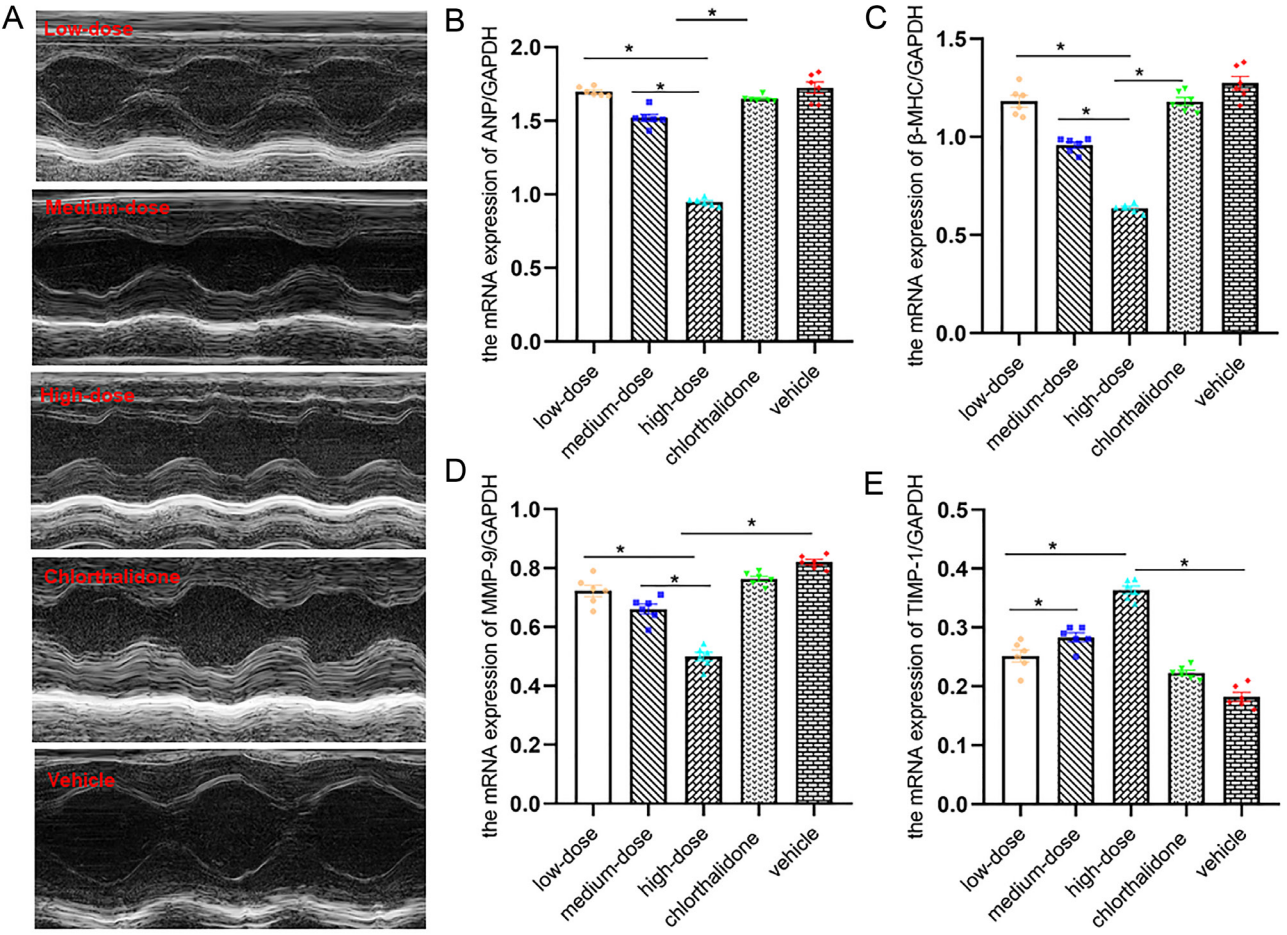
mTOR inhibitor rapamycin attenuated testosterone-induced OVX SHR myocardial hypertrophy and hypertrophy-related gene expression. (A) mTOR inhibitor rapamycin abolished the effects of testosterone-induced OVX SHR myocardial hypertrophy. (B, C, D, and E) mRNA expression of ANP, β-MHC, MMP-9 and TIMP-1 by real-time RT-PCR. Data shown are expressed as mean ± S.E.M. The *P* values were determined by one-way ANOVA (*n* = 6 independent biological samples). *P < 0.05. MHC, myosin heavy chain; MMP, matrix metalloproteinase; TIMP, the tissue inhibitor of metalloproteinase.

The high-dose rapamycin group showed significant reductions in the HW/TL level, whereas the low-dose and medium-dose rapamycin groups had no significant effect on the HW/TL level. Compared with vehicle group, HW/TL and HW/BW in other groups were decreased. There was no difference in UW/TL, T and E levels in each group (Table 1). ELISA results showed that there was no difference in urinary microalbumin among all groups (Supplementary Fig. 1). LVM in the high-dose group (263.12 ± 3.20 g) was reduced compared with the other four groups (low-dose 440.59 ± 16.83 g vs medium-dose 350.78 ± 17.54 g vs chlorthalidone 443.86 ± 20.17g vs vehicle 530.51±10.98g). There was no difference in the levels of IVST and LVPWT between low-dose and medium-dose groups. Compared with the other four groups, LVM, LA, E/A and E/e’ were higher than vehicle group (Fig. 5A and Table 2). Compared with low-dose and medium-dose groups, the area of cardiomyocytes decreased in the high-dose group. The area of cardiomyocytes in vehicle group was increased compared with that in the other four groups. The area of cardiomyocytes in chlorthalidone group was larger than that in medium-dose group (Fig. 4B and D).

The analysis of these effects showed that the mRNA expression levels of ANP, β-MHC and MMP-9 reduction were greater in the high-dose rapamycin group compared to the low-dose and the medium-dose group and did not change in the chlorthalidone group (Fig. 5B, C and D). The mRNA expression levels of TIMP-1 in myocardial tissue of vehicle group were significantly lower than those of the other four groups (Fig. 5E).

### Rapamycin attenuated the effect of T on mTOR/S6K1/4EBP1 signaling pathway

**Figure 6:**
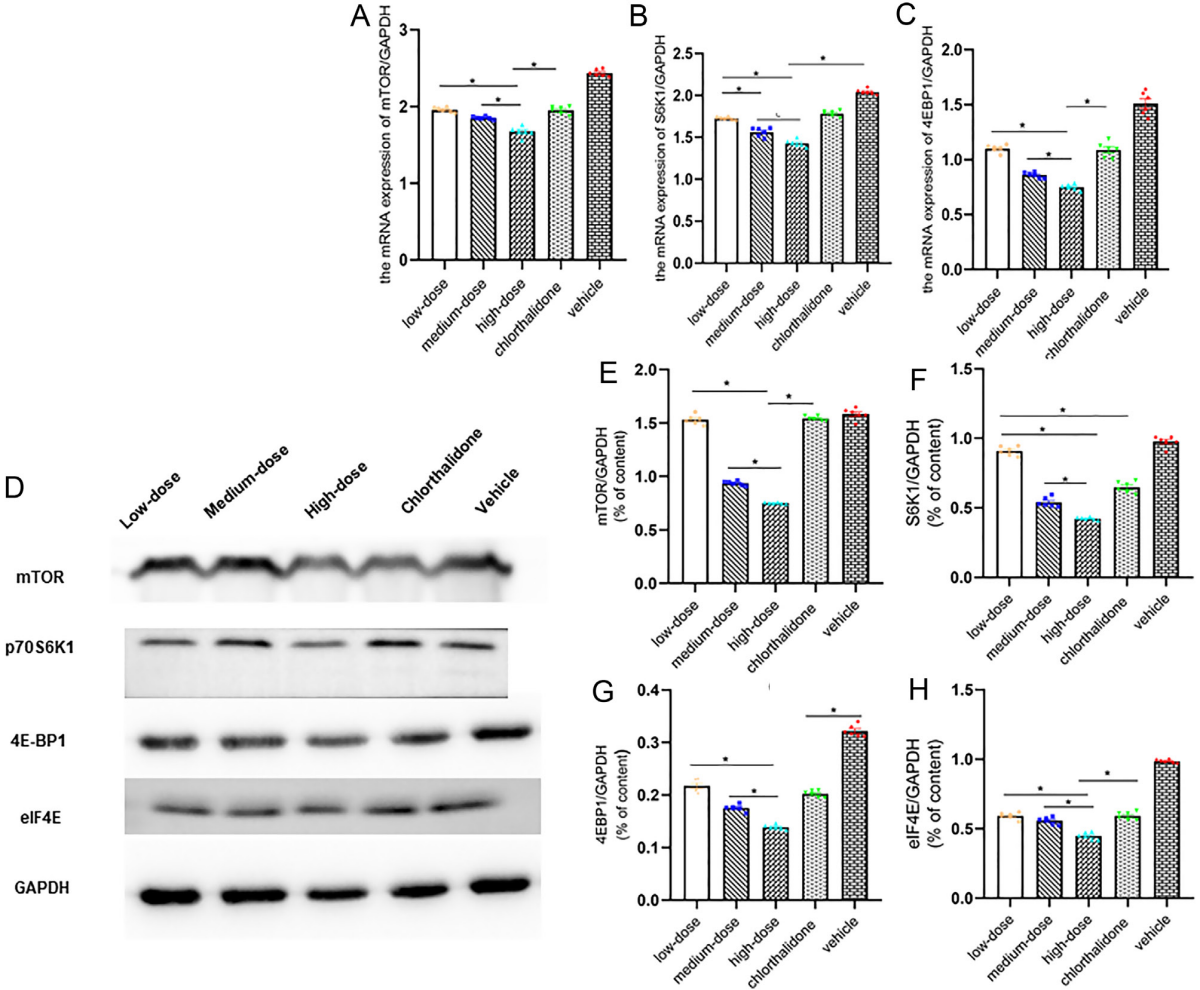
mTOR inhibitor rapamycin alleviated the effect of testosterone on mRNA and protein expression of mTOR signaling pathway in myocardial tissue. (A, B and C) Rapamycin attenuated the effect of testosterone on mRNA expression of mTOR signaling pathway. (D, E, F, G and H) Rapamycin alleviated the expression of mTOR signaling pathway proteins. Data shown are expressed as mean ± S.E.M. The *P* values were determined by one-way ANOVA (*n* = 6 independent biological samples). *P < 0.05. eIF4E, eukaryotic translation initiation factor 4E; mTOR, mammalian rapamycin receptor; S6K1, ribosomal protein S6 kinase; 4EBP1, 4E binding protein 1.

The expression levels of mTOR, S6K1, 4EBP1 and eIF4E in myocardial tissue of high-dose group were lower than those of low-dose and medium-dose group (Fig. 6D, E, F, G and H). As for the protein and mRNA expression levels of mTOR, S6K1 and 4EBP1 in myocardial tissue, vehicle group presented a significant increase in relation to the other groups (Fig. 6A, B, C, D, E, F and G). mTOR and 4EBP1 protein and mRNA expression in chlorthalidone group was paralleled with the low-dose group in myocardial tissue (Fig. 6A, C, D, E and G).

## Discussion

The main finding of this study is that testosterone, at physiologically relevant concentrations, leads to the development of female SHR LVH after ovariectomy with associated increase in (1) left ventricle: cardiomyocyte area and LVMI, HW/TL; (2) left ventricular myocardial tissue mTORC1, S6K1, 4EBP1, ANP, β-MHC and MMP-1 expression; (3) rat caudal artery SBP and DBP. A major finding in our study is that testosterone-mediated changes in the expression level of myocardial mTOR/S6K1/4EBP1/eIF4E signaling pathway play a critical role in the pathogenesis and development of cardiac hypertrophy in SHR after ovariectomy. In addition, we found that rapamycin, an mTORC1 inhibitor, can delay the occurrence of myocardial hypertrophy in testosterone-induced SHR after ovariectomy on the basis of antihypertensive therapy. More importantly, our research results suggest that the protective effect of mTORC1 inhibitor is correlated with the application dose, that is, the protective effect of mTORC1 inhibitor on testosterone-induced myocardial injury in rats may be in the range of 1.5–2 mg/kg.

It is well known that the occurrence of hypertension is complex and associated with multiple systemic changes (Schütten *et al.* 2017, Drummond *et al.* 2019). Sex hormones play an important role in regulating blood pressure in both men and women (Jiménez *et al.* 2018, Hester *et al.* 2019). Androgens, especially testosterone, play a key role in cell growth (Hanson *et al.* 2020, Stone & Stachenfeld 2020). The characteristics of testosterone synthesis and metabolism involve increased protein synthesis, which is essential for normal and hypertrophic growth of cardiomyocytes (Carbajal-García *et al.* 2020, Troncoso *et al.* 2021). In the present study, ovariectomized SHR decreased blood pressure (mean decrease of 4 mmHg) and improved myocardial hypertrophy after estrogen supplementation. However, testosterone intervention induced increased blood pressure (mean increase of 28 mmHg) and cardiac hypertrophy had a greater effect. These findings indicate that T plays an important role in elevated blood pressure and cardiac hypertrophy in ovariectomized SHR.

Testosterone-induced ovariectomy SHR cardiac hypertrophy may be related to the expression of mTORC1/S6K1/4EBP1/eIF4E. mTOR participates in regulating cell protein synthesis and biosynthesis by sensing and integrating different upstream stimulus signals (Gu *et al.* 2020, Lv *et al.* 2020). mTORC1 activates S6K1 and 4EBP1 in parallel (Borack *et al.* 2021). The activation of S6K1 and 4EBP1 can alter the protein translation dynamics and accelerate the translation process (Patra *et al.* 2021). eIF4E is a 4EBP1 downstream regulator that can bind to eIF4G to form the translation initiation complex eIF4F. Therefore, the expression level of eIF4E has become the key point for controlling protein translation and expression (Cope *et al.* 2014). Previous studies have shown that mTOR/p70S6K1 signaling pathway is necessary for the cardiomyocyte hypertrophic response induced by angiotensin II or phenylephrine (Li *et al.* 2018, Liu *et al.* 2021). However, the growth of the heart* in vivo* is a more complicated process caused by a combination of many factors. Our results showed that the myocardial hypertrophy of Testosterone-mediated OVX SHR was accompanied by a significant increase in mTOR and downstream targets S6K1, 4EBP1 and eIF4E protein levels. Therefore, this study provides evidence that mTOR/S6K1/4EBP1/eIF4E signaling pathway may be necessary for the mechanism of the Testosterone-induced OVX SHR myocardial hypertrophy response.

It is worth noting that there are inconsistent reports on the effect of ovariectomy on blood pressure. Loh and Salleh (2017) found that the mean arterial pressure of 8-week-old female SHR decreased after gonadectomy. Masubuchi *et al.* also showed that gonadectomy may affect blood pressure in adult female rats (Masubuchi *et al.* 1982). In contrast, other studies reported that ovariectomies did not cause changes in blood pressure in WT mice (De Melo *et al.* 2016, Pijacka *et al.* 2016). No differences in blood pressure levels were found between intact and ovariectomized Sprague–Dawley rats aged 10–12 weeks (Xue *et al.* 2009). Consistent with these results, our results also indicated that there was no significant change in blood pressure in ovariectomized SHR compared with sham-operated group. We speculate that the possible reasons for the above phenomenon are as follows: (1) gonadectomy in rats of different ages causes different responses of cardiovascular system to changes in the levels of sex hormone axis level; (2) ovariectomy itself may not be directly related to the regulation of blood pressure but is caused by dynamic changes in the level of sex hormones; and (3) aromatase activity and sex hormone binding globulin levels secreted by peripheral tissues (such as liver, fat, muscle, etc.)* in vivo* after OVX affects sex hormone levels, thus leading to inconsistent blood pressure levels.

The mTORC1 inhibitor rapamycin forms a complex with FK506 binding protein to inhibiting activation of S6K1 and 4EBP1 downstream signals (Abe *et al.* 2019). Partial inactivation of mTOR caused by genes or drugs can inhibit pathological hypertrophy of the myocardium maintaining the heart’s adaptability to stress overload (Oeing *et al.* 2020). The specific knockout of rheb1 gene in mice under pressure overload can inhibit the expression of mTORC1 and reduce the occurrence of myocardial hypertrophy and myocardial fibrosis (Wu *et al.* 2013). On the one hand, the reduced expression of ANP and β-MHC in this study suggests that the reversal of cardiac hypertrophy after rapamycin treatment is not only due to the inhibition of mTOR but also fundamentally changes the pathological properties of the hypertrophy (such as the increase of wall stress). On the other hand, changes in the expression levels of the S6K1 and 4E-BP1 protein confirmed that rapamycin did block mTOR signaling transduction in the heart tissue. Therefore, mTORC1 may become an important regulator and potential new target of androgen hormone metabolism signal/cardiac hypertrophy.

MMPs can cause myocardial interstitial fibrosis and loss of contractile function by degrading matrix components and increasing abnormal collagen synthesis (Cabral-Pacheco *et al.* 2020). The MMPs/TIMPs system balance contributes to the regulation of collagen synthesis and degradation and plays an important role in maintaining the structure and function of the heart (Pei *et al.* 2010). In the present study, the high expression of MMP-9 and the low expression of TIMP-1 displayed imbalance in the Testosterone-induced cardiac hypertrophy of OVX SHR. More importantly, our results suggested that rapamycin alleviated the dysregulation of MMP-9/TIMP-1 balance while improving Testosterone-induced OVX SHR cardiac hypertrophy.

Rapamycin effectively reduced Testosterone-induced myocardial hypertrophy at clinically relevant doses, which is related to the attenuation of the increased myocardial cells size. In this study, low, medium and high doses of rapamycin have the effect of reversing Testosterone-induced SHR myocardial hypertrophy after OVX under the premise of the same administration method, but the high-dose administration group has the best effect. These results indicated that the myocardial protective effect of rapamycin had a certain correlation with the applied dose. In addition, the protective effect of mTORC1 inhibitor on testosterone-induced myocardial injury in rats may be in the range of 1.5–2 mg/kg. The dose-dependent and reversible side effects of rapamycin have been found in the large number of clinical trials. In this study, the application of the maximum dose of rapamycin did not find significant renal damage. Considering that the treatment of myocardial hypertrophy by rapamycin is not proportional to the degree of high blood pressure overload damage, we used echocardiography to evaluate LVEF and LVFS as measures of cardiac function. In this study, our data showed that the ejection fraction and the shortening fraction were similar in the treatment of cardiac hypertrophy by injection with different doses of rapamycin. Furthermore, rapamycin also did not cause fatal events or weight loss in rats.

Some limitations in this study should also be considered. First, this study effectively identified the mTOR signaling pathway as a potential target of testosterone-induced OVX SHR cardiac hypertrophy, but it did not explore mTOR upstream regulatory molecules. Future research design can further identify the upstream regulators and mechanisms to comprehensively elucidate the role of testosterone in OVXSHR cardiac hypertrophy. Furthermore, ELISA may not be the most sensitive means to detect hormone levels. In the future, the highly sensitive and specific liquid chromatography-tandem mass spectrometry methods can be used to detect plasma testosterone levels. Finally, the relationship between the total elevated levels of these proteins (mTOR, S6K1 and 4E-BP1) induced by testosterone and their phosphorylated form remains unclear and requires further investigation.

However, these findings have at least three important clinical implications. First, androgen may play an important role in promoting myocardial hypertrophy in postmenopausal hypertensive women. Therefore, it also partially explains the reasons why blood pressure or other cardiovascular event risks have not been improved even after estrogen supplementation in many major clinical studies. Secondly, it was confirmed that mTORC1/S6K1/4E-BP1 signaling pathway is an important pathway for testosterone-induced myocardial hypertrophy in postmenopausal hypertensive female rats. Our findings aim to elucidate the cellular basis for increased relative tendency of hypertension and left ventricular hypertrophy in postmenopausal women and serve to help open up new research pathways. Finally, it is clear that rapamycin reversed the effect of myocardial hypertrophy under specific conditions and further screened the optimal dose. We also hope to find specific targets for target organ protection on the basis of antihypertensive therapy and provide a new treatment idea for postmenopausal women with hypertension and LVH. In conclusion, this study demonstrates that the regulation of mTOR/S6K1/4E-BP1 signaling pathway may be one of the important mechanisms for the occurrence of myocardial hypertrophy in testosterone-induced OVX SHR. Moreover, rapamycin specifically identifies and blocks testosterone-induced OVX SHR cardiac hypertrophy through mTOR signaling pathway. These results indicated that the mTOR pathway plays a key role in testosterone-induced OVX SHR myocardial hypertrophy. Therefore, on the basis of antihypertensive therapy, mTOR inhibitors may provide a new therapeutic candidate for delaying myocardial remodeling and cardiac insufficiency in postmenopausal hypertensive women.

## Supplementary Material

Supplementary figure 1. Representative Vaginal smear results on the 7th day of each model (original magnification×400).

Supplementary figure2. Urine microalbumin levels of rats after mTOR inhibitor intervention Data shown are expressed as mean ± S.E.M. The p values were determined by one-way ANOVA (n=6 independent biological samples). *P < 0.05.

## Declaration of interest

The authors declare that there is no conflict of interest that could be perceived as prejudicing the impartiality of the research reported.

## Funding

This study was supported by the National Natural Science Foundation of China http://dx.doi.org/10.13039/501100001809 (NSFC 81670385), Gansu Province Health Research http://dx.doi.org/10.13039/100005622 Project (GSWSKY2017-02), and Cuiying Scientific and Technological Innovation Program of Lanzhou University http://dx.doi.org/10.13039/100012899 Second Hospital (CY2017-QN09).
