# Supplementary figures and images for: mTOR inhibitor improves testosterone-induced myocardial hypertrophy in hypertensive rats
Source: J Endocrinol. 2021 Dec 6;252(3):179–93. doi: 10.1530/JOE-21-0284 (PMC8859925; doi:10.1530/JOE-21-0284)

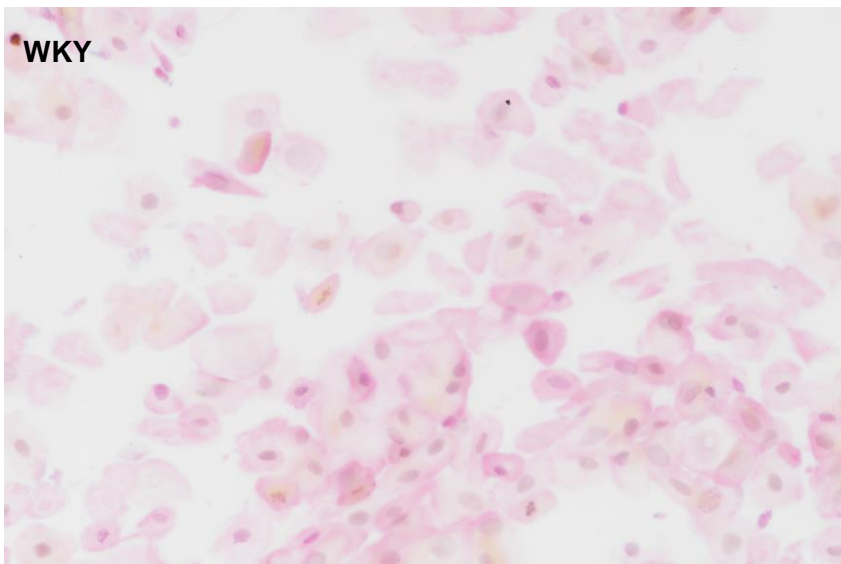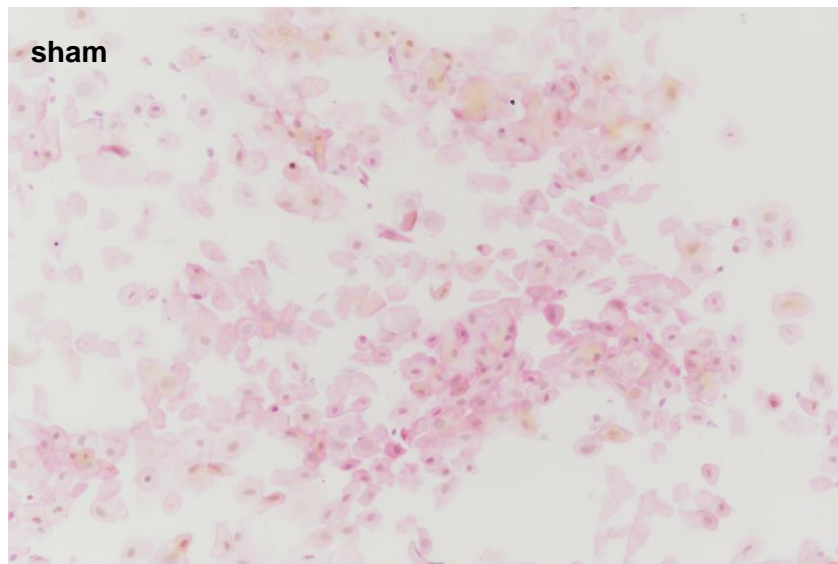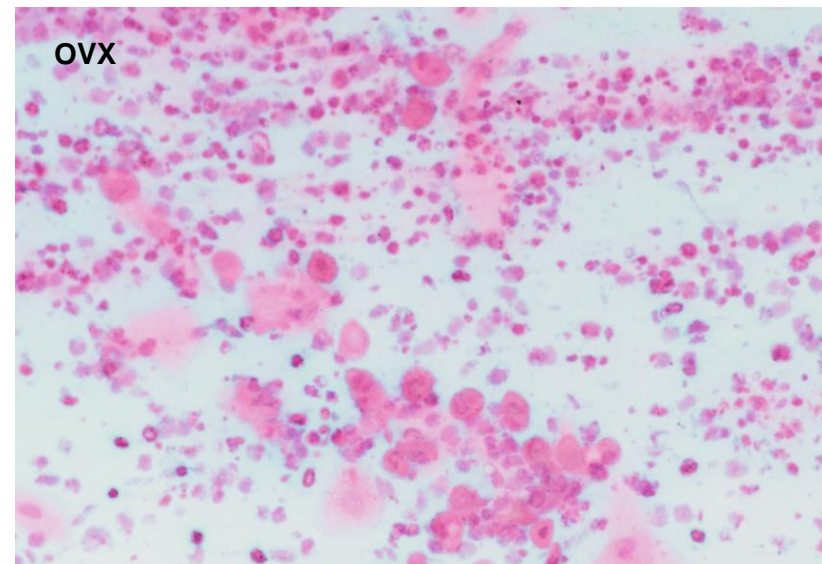

Supplement: Supplementary figure 1. Representative Vaginal smear results on the 7th day of each model (original magnification×400). [file supplementary_figure_1.pdf]

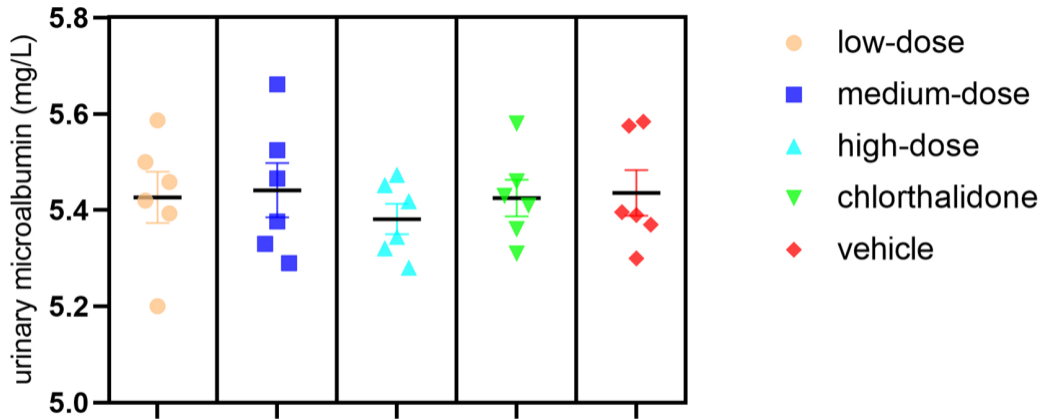

Supplement: Supplementary figure2. Urine microalbumin levels of rats after mTOR inhibitor intervention Data shown are expressed as mean ± S.E.M. The p values were determined by one-way ANOVA (n=6 independent biological samples). *P < 0.05. [file supplementary_figure_2.pdf]
